# Trypanosomatid species infecting bats in the Fiocruz Atlantic Forest Biological Station, an urban forested fragment in Rio de Janeiro, Brazil

**DOI:** 10.1371/journal.pone.0349312

**Published:** 2026-05-18

**Authors:** Alice Pereira Berbigier, Juliana Helena da Silva Barros, Roberto Leonan Morim Novaes, Ricardo Moratelli, Bruno Alves, Cristiane Varella Lisboa, Ana Maria Jansen, André Luiz Rodrigues Roque

**Affiliations:** 1 Fundação Oswaldo Cruz, Instituto Oswaldo Cruz, Laboratório de Biologia de Tripanosomatídeos, Rio de Janeiro, Brazil; 2 Fundação Oswaldo Cruz, Fiocruz da Mata Atlântica, Rio de Janeiro, Brazil; Fiocruz Minas: Fundacao Oswaldo Cruz Instituto Rene Rachou, BRAZIL

## Abstract

Trypanosomatids (Protozoa; Kinetoplastea) are obligate parasitic protozoans that infect a wide range of vertebrate hosts, including bats – the only volant mammals, widely distributed across the globe. In Rio de Janeiro, Brazil, Atlantic Forest remnants persist within urban landscapes, including the Fiocruz Atlantic Forest Biological Station (EFMA), a unit embedded in a highly urbanized environment. Between 2013 and 2019 (excluding 2015), field surveys were conducted in four landscape physiognomies with different degrees of environmental degradation. Blood and other tissue samples (spleen, liver and skin) were collected from bats to investigate the infection and diversity of trypanosomatid infections. A total of 270 bats were examined through parasitological techniques (fresh blood examination and hemoculture) and molecular diagnosis through phylogenetic analysis. Overall, 24.1% (n = 65) of bats tested positive for trypanosomatids. Molecular identification revealed a diverse assemblage of species, including *Crithidia mellificae*, *Trypanosoma cruzi* (DTUs TcI and TcII), *Trypanosoma dionisii*, *Trypanosoma* sp. Neobat 1 and 4, *Trypanosoma lainsoni,* and *Trypanosoma rangeli* A. Our findings underscore the critical role of direct molecular detection in tissue samples for uncovering infections missed by traditional parasitological methods, particularly those caused by uncultivable species/genotypes such as *Trypanosoma* sp. Neobat 1 and 4. The recurrent detection of *T. dionisii*, *T. cruzi* DTU TcI and *T. rangeli* A, reinforces their sustained circulation in bats from this urban-forest interface. Moreover, the presence of *C. mellificae*, traditionally regarded as a monoxenous species, adds new bat hosts to the already extant list of mammal species recognized as able to harbor this parasite. Notably, this study provided the first record of *T. lainsoni* and *T. cruzi* DTU TcI in mammals from the EFMA, highlighting a surprising richness of trypanosomatids in this small, and human-impacted forest remnant. The high diversity of trypanosomatid communities infecting bats, recognized as habitat-resilient hosts, reinforces the complexity of the sylvatic transmission of those parasites that may not be disrupted even under strong anthropogenic pressures.

## Introduction

Kinetoplastea encompasses free-living flagellated protozoa and obligate parasites [1]. This class includes the order Trypanosomatida and its unique family, Trypanosomatidae, which is composed of obligatory parasites from vertebrates, invertebrates and plants, and whose transmission cycles may be monoxenic or heteroxenic, depending on one or at least two hosts to complete its life cycle, respectively [2,3]. These boundaries between monoxenous and heteroxenic species are quite fuzzy, as increasingly recognized in recent studies of mammal species infected by putative monoxenic parasites. *Leptomonas seymouri* was observed parasitizing immunocompromised humans in mixed infection with *Leishmania donovani* [4], while *Crithidia mellificae*, a common bee parasite, was isolated from a nectarivorous bat, primates and carnivores [2,5].

The most studied genus present in the Trypanosomatidae is *Trypanosoma*, which comprises several species of vertebrate and invertebrate parasites, including some of them of medical and veterinary importance, such as *Trypanosoma cruzi,* the etiological agent of Chagas disease, which has therefore been widely studied in Americas [6]. One of the most puzzling traits of *T cruzi* is its huge genetic heterogeneity that is grouped into lineages termed Discrete Typing Units (DTU), named as TcI to TcVI, and TcBat [7,8]. Another trypanosomatidae species which also has genetic heterogeneity is *Trypanosoma rangeli,* heteroxenous parasite from mammals and kissing bugs (Reduviidae; Triatominae) from Americas. *Trypanosoma rangeli* is also genetically diverse and five lineages have been described so far, named as TrA to TrE [9].

Bats are some of the most ancient trypanosomatid hosts in which a huge species richness was already demonstrated, as *T. cruzi*, *T. rangeli*, *Trypanosoma dionisii*, and the genotypes *Trypanosoma sp*. Neobat 1–6 [10–12]. *Trypanosoma dionisii* is a biological and genetic heterogeneous parasite that was considered specific to bats for a long time, but recently described in humans, marsupials, rodents and carnivores [13–16]. The *Trypanosoma* Neobat genotypes are Molecular Operational Taxonomic Units (MOTUs) phylogenetically close to *T. wauwau,* and represent another bat restricted *Trypanosoma,* but not yet formally described as species [10–12]. In addition, the currently recognized *T. cruzi* clade, in which all these parasites are included, is believed to have evolved from an ancestral bat trypanosome, supporting the “Bat Seeding Hypothesis” [17]. Bats comprise a cosmopolitan and well-adapted group of organisms found in different environments, as is the case of the Brazilian Atlantic Forest, where more than 100 species are recorded [18]. In this ecoregion, it was already described different bat species infected by trypanosomatids in environments displaying completely different characteristics [5,11,13].

There are several methodologies used to diagnose trypanosomatid infections in mammals. Parasitological diagnoses, such as fresh blood examination, xenodiagnoses and hemoculture despite its low sensibility, when positive, demonstrate expressive parasitemia, consequently, the potential of a host to be a source of infection for vectors. Besides, hemocultures also enables parasite isolation and morphological and biochemical analyses possible. Molecular diagnosis directly in host tissues can detect lower parasite loads, including parasite subpopulations that do not grow in the most employed culture media [13,19]. The use of both approaches enables the detection of a greater trypanosomatid diversity in different hosts [19].

The Pedra Branca Forest (Parque Estadual da Pedra Branca) is the largest urban forest in the world, comprising a remnant of 12,000 ha of Atlantic Forest in the metropolitan zone of Rio de Janeiro municipality, Brazil [20]. Several protected areas and initiatives for the environment conservation of Pedra Branca Forest occur and one of them is the Fiocruz Atlantic Forest Biological Station (Estação Biológica Fiocruz Mata Atlântica - EFMA), a biological station with a low-income population and vegetational cover ranging from Periurban to well-preserved forest, where several parasitological studies in the local fauna and plants have already been carried out [19,21]. In the EFMA, trypanosomatid infections were described in bats, rodents, marsupials, and humans, including the description of a new species, named *Trypanosoma janseni* [5,19,22,23], demonstrating that even this urban and highly anthropized area can reveal a wide, and sometimes unknown, diversity of trypanosomatid species [19]. Considering that bats are hosts with a wide capacity for movement and considerable longevity and interact with different vertebrate and invertebrate hosts of trypanosomatids, these flying mammals certainly contribute to the maintenance and dispersal of trypanosomatids in various habitats. Moreover, bats are considered ancestors of trypanosomes of the *T. cruzi* clade, whose diversity is still poorly known [5,10–12], and these phylogenetic and ecological characteristics shed light on this topic and led us to investigate the diversity of trypanosomatids in bats of an urban remnant of the Atlantic Forest. In this study, we conducted the diagnosis of trypanosomatid infection in bat species from EFMA captured during six years in different sampling sites according to their level of degradation. From these, tissue cultures and molecular diagnoses were carried out.

## Methods

### Study area

This study was conducted in the Fiocruz Atlantic Forest Biological Station (EFMA) in Fiocruz, Rio de Janeiro, Brazil (22^°^56’23” S, 43^°^24’13” W, 20 m a.s.l.), between 2013 and 2019 (excluding 2015). The animals included in this study were captured as part of different research projects conducted by multiple partners in the same locality over the seven-year sampling period. Due to this dynamic, sampling effort was equivalent across areas in total, but variable across years or sampling periods; therefore, no ecological analyses involving bat capture dynamics or parasite prevalence and species richness were performed. The EFMA is administered by Fundação Oswaldo Cruz (Fiocruz) since 2016 and this environment is composed of secondary dense ombrophilous forest, including preserved and degraded areas with the presence of low-income communities, where several interdisciplinary research and actions occur, such as environmental health, urban planning, and public policies [20,23,24].

**Fig 1:**
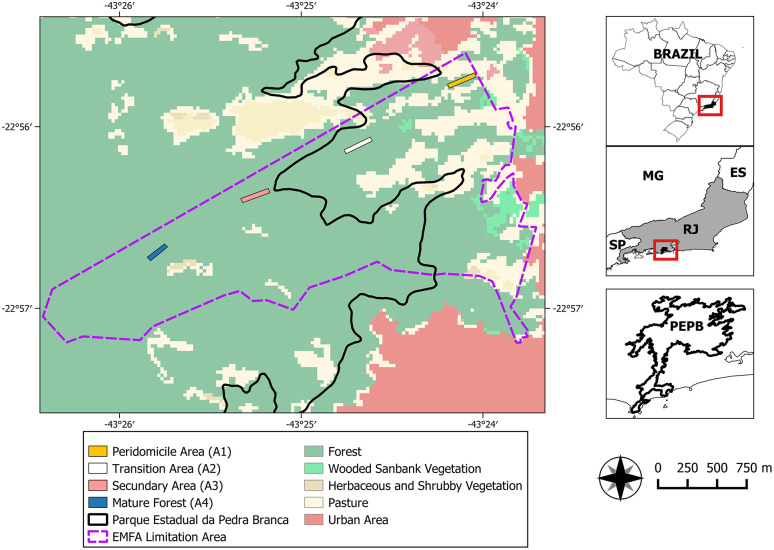
Sites of bat sampling in EFMA, Rio de Janeiro, Brazil.The map was done in software QGIS 3.22.7 with the use sand from MapBiomas, delimitations of Brazil from IBGE (Instituto Brasileiro de Geografia e Estatística), and EFMA delimitations from EFMA team.

The bat captures were performed in four areas according to distinct levels of human disturbance (Fig 1): 1. Peridomicile (A1): representing areas adjacent to human dwellings with high anthropogenic action and vegetation dominated by exotic plant species; 2. Initial Secondary Forest (A2): disturbed forest in early process of regeneration with low plant diversity and vegetation dominated by ruderal arboreal shrub species; 3. Late Secondary Forest (A3): secondary forests in different stages of regeneration with an ombrophilous dense vegetation, including the presence of canopy greater than 15 m and large abundance of epiphyte bromeliads; and 4. Mature Forest (A4): the most preserved and distant area from the human dwellings, with a canopy reaching 20 m high and a very diverse native flora, including palm trees, hardwood trees, epiphyte bromeliads and orchids [24,25].

### Bat capture and identification

Captures of bats were carried out over 36 nights, as follows: 5 nights in 2013 in A1, A2 and A3, 8 nights in 2014 in A1, A2 and A3, 2 nights in 2016 in A4, 11 nights in 2017 and 9 in 2018 in the 4 areas, and 1 night in 2019 in A2, with equal sampling effort for all sites (Table 1). Bat captures were performed using 10 mist-nets/night (9 × 3 m, 20 mm mesh) per area that were placed in clearings in the vegetation, along trails, over water bodies and near flowering or fruiting plants. Mist-nets were opened at sunset and closed after four hours. Captured animals were kept in cotton bags and individuals were identified by external and cranial features using morphological characters [25]. The animals were transported by foot to the field laboratory and processed in the following morning. Pregnant and lactating females, besides the individuals captured that exceed the number of authorized in our licenses, were immediately released in the same capture points. Sampling effort was calculated by multiplying the number of mist nets by the net area (height × length), the number of sampling days, and the number of hours that the nets remained open following (Table 1) [26].

**Table 1:** Sampling effort (in m².h) and total of sampling nights (in parentheses) applied for captures of bats in different sites from Fiocruz Atlantic Forest Biological Station, Rio de Janeiro.

Sampling sites	2013	2014	2016	2017	2018	2019	Total
A1 Peridomicile	2,700 (2)	4,050 (3)	–	1,350 (1)	4,050 (3)	–	12,150
A2 Initial Secondary Forest	2,700 (2)	4,050 (3)	–	2,700 (2)	1,350 (1)	1,350 (1)	12,150
A3 Late Secondary Forest	1,350 (1)	2,700 (2)	–	5,400 (4)	2,700 (2)	–	12,150
A4 Mature Forest	–	–	2,700 (2)	5,400 (4)	4,050 (3)	–	12,150
**Total**	6,750 (5)	10,800 (8)	2,700 (2)	14,850 (11)	12,150 (9)	1,350 (1)	4,8600

### Field procedures

In a field laboratory set exclusively for this purpose, the captured bats were anesthetized by a combination of ketamine (100 mg/ml) and acepromazine (10 mg/ml), in a 9:1 proportion (0.15 ml/100g of individual collected) and blood was collected through cardiac puncture for cultures and fresh blood examination. The euthanasia of captured individuals was performed using 19.1% potassium chloride intracardially (2 mg/kg of the individual) and spleen, skin and liver tissues were collected for culture and molecular analysis [27].

For fresh blood examination a drop of approximately 5 µL of blood was placed between the slide and coverslip and observed in optical microscope under 400x of magnification. Samples with the presence of flagellates forms compatible with trypanosomatids were considered positive. For hemoculture, approximately 0.6–0.8 mL of blood from each animal was divided into two tubes containing NNN (Nicolle, Novy, and McNeal) and LIT (Liver Infusion Triptose) or Schneider liquid culture medium (one each) [28].

Tissues fragments were stored either in sterile saline solution, antibiotics, and antifungals for culture (sodium chloride-NaCl at 58.44 g/mol containing 10 mg streptomycin, 25 µL amphotericin B, and 10,000 IU penicillin per mL, Sigma™ commercial solution, St Louis, MO, USA), or in absolute ethanol that was stored in a freezer at −20^o^C for subsequent molecular diagnosis [29].

### Culture monitoring and DNA extraction

Hemocultures were observed every two weeks for five months, while tissue samples were maintained in saline solution at 4^o^C for 24 h and transferred to culture tubes containing NNN medium and Schneider liquid medium, then observed twice a week for one month [27,28]. Blood cultures were performed to isolate *Trypanosoma* sp., which grows more slowly in culture medium and can therefore be monitored every two weeks. Because it is isolated from blood, a tissue normally free of bacterial contamination, its monitoring can be carried out for up to five months. Tissue cultures, on the other hand, were monitored twice a week because they aimed to isolate *Leishmania* sp., which grows faster in culture medium. The fact that it is examined more frequently and originates from non-sterile tissues, and whose collection procedures are more susceptible to contamination, prevents monitoring the cultures for more than one month.

When positive, the parasite population was expanded (by adding the same liquid culture medium) until it reaches approximately 10⁸ parasites when the culture was centrifuged for DNA extraction. Parasite expansion was performed whenever the parasite population showed reduced motility compared to the previous observation. Except for one sample (LBCE 10399), no bacterial contamination was observed in the cultures that influenced parasite isolation or detection rates. Samples from positive cultures had their DNA extracted using Qiagen— Dneasy Blood and Tissue Genomic Kit™ (Hilden, North Rhine-Westphalia, Germany) following the manufacturer’s instructions and stored in a freezer at −20^o^C until use.

### Molecular detection and sequencing

Spleen, skin, and liver fragments collected in ethanol were previously rehydrated (by washing three times with Milli-Q water) to remove ethanol and subjected to DNA extraction using the same Qiagen kit or the Wizard Genomic DNA Purification Kit (Promega, Madison, WI, USA). The extracted DNA was stored in a freezer at −20^o^C until use. Experimentally infected and uninfected hamster tissues were used as positive and negative controls in DNA extraction from bat tissues and the same controls, besides another negative (ultrapure water) and positive (*T. cruzi* DNA) controls were also used in PCR reactions. For positive cultures we included only negative control (ultrapure water) in DNA extraction because these samples were already known to be positive and included positive control (*T. cruzi* DNA) in PCR reactions, besides ultrapure water as negative control, to evaluate the PCR reagents.

Molecular diagnoses were performed applying a Nested PCR targeting the partial region of the 18S rDNA (approximately 600 bases pairs) and conducted according to Smith *et al.* (2008) [30] with the following modifications: a final volume of 25 µL was used, containing 13.5 µL of ultrapure water, 8.5 µL of GoTaq Master Mix (Promega, Madison, WI, USA), and 2 µL of DNA in both rounds. In the first round, 0.5 µL of the primers TRY R and F (16 pmol) (Eurofins Genomics™, Val Fleuri, Luxembourg, Luxembourg) were used, and in second round, 0.5 µL of primers SSU R and F (16 pmol) (Eurofins Genomics™, Val Fleuri, Luxembourg, Luxembourg). Cycling condition was defined with an initial denaturation at 94^o^C for 3 min, 94^o^C for 30 s, 55^o^C for 1 min, 72^o^C for 1.5 min, 94^o^C for 30s (29x), 72^o^C for 10 min and 4^o^C when finished, in a Swift™ Max Pro Thermal Cycler 16 thermal cycler (model SWT-MXP-BLC-1) or BIO – RAD Thermal Cycler (model T100™). *T. cruzi* DNA (Y strain) was used as positive control, and ultrapure water was used as negative control in each reaction.

Electrophoresis was realized to visualize the amplified products in a 2% TBE (Tris-Borato-EDTA) agarose gel and stained with GelRed Biotium. The gels were visualized on the UV Transilluminator 2000 (BIO-RAD) or in Gel Logic 212 Pro photo documenter using the Carestream MISE program, using a molecular weight marker of 100 base pairs (bp) as a reference (Ludwig Biotecnologia, Alvorada, Rio Grande do Sul, Brazil).

Positive products were purified with Illustra™ GFX™ PCR DNA/Gel Band Purification kit (GE Healthcare, Chicago, IL, USA) or Wizard SV Gel kit and Clean-up System PCR kit (Promega, Madison, WI, USA) following the manufacturer’s instructions. The products were sequencing by Sanger method with the BigDyeTM Terminator v3.1 Cycle Sequencing Ready Reaction Kit (Applied Biosystems, Waltham, MA, USA) in an ABI3730 DNA Analyzer Automatic Sequencer (Applied) at PDTIS/FIOCRUZ Sequencing Platform.

The positive cultures were amplified and cryopreserved in the *Trypanosoma* Collection of Wild and Domestic Mammals and Vectors (Coleção de *Trypanosoma* de Mamíferos Silvestres, Domésticos e Vetores – ColTryp). Positive cultures that were not able to grow and amplify in culture medium were centrifuged to obtain sediments. The sediments were extracted using phenol–chloroform method [31] and submitted to the Nested PCR above mentioned. Moreover, we had one specific case (LBCE 10399) in which two characterizations were performed, one using the culture sediment and another using parasites re-isolated from blood of a mouse intraperitonially inoculated with parasites from the primary culture. This procedure was performed due to heavily bacterium contamination in the primary culture and aimed to re-isolate the parasites from blood mice during patent parasitemia. For this procedure, two male Swiss mice aging 6 weeks were intraperitonially inoculated with 10^7^ flagellates from primary culture and followed up each 48h by examining the presence of flagellates in a drop of blood from mice tails (direct observation in 400x microscopy). Once positive (about one week), the unique positive mouse was submitted to hemoculture as described for bats.

### Phylogenetic analysis

To check the quality and size of the sequences, the BioEdit Sequence Alignment Editor program was used [32]. The consensus sequences obtained were manually edited using the SeqMan-DNA Star Program [33] and compared for similarity with sequences deposited in GenBank database from the National Center for Biotechnology Information (NCBI) using the BLAST algorithm (Basic Local Alignment Search Tool). For species identification, the following values were adopted: cover (≥99%), identity (≥99%), and E-value (0.0).

Three phylogenetic trees were constructed by maximum likelihood (ML) from 18S rRNA gene sequences obtained in this study, which were aligned in MEGA 11 by the MUSCLE algorithm [34]. The same phylogenetic tree model was used for the three trees, according to the best model test generated by MEGA 11: Kimura 2-parameter model and Gamma distribution to model evolutionary rate differences among sites (4 categories (+G, parameter = 0.2566)). The percentage of trees on which the associated taxa clustered together is shown next to the branches of the trees. To branch support, ultrafast bootstrapping of 5,000 replications with 1000 maximum interactions was applied [35].

Bayesian analysis (BI) was also performed using the PhyloSuite program [36], using MrBayes [37] to define the best phylogenetic tree model, which was: GTR (Generalized Time-Reversible) and Gamma (+ G, Gamma-distributed variation) for the three trees generated in this study. The Bayesian Markov Chain Monte Carlo (MCMC) method was used, where four runs and chains were performed for 20 million generations.

The ML and BI trees were concatenated, and representative sequences were used for the construction of phylogenetic trees, along with representative sequences of trypanosomatid species observed in this study.

### Statistical analysis

The trypanosomatid prevalence rates were calculated for each capture area as the proportion of infected bats in any of the parasitological/molecular assays, in relation to total number of examined. Chi-square contingency tests were performed to assess differences in the number of animals captured and infected in the different capture areas. These analyses were performed using basic functions implemented in R software [38] considering a significance level of 95%.

### Ethics statement

The sampling procedures were authorized by IBAMA (Instituto Brasileiro do Meio Ambiente e dos Recursos Naturais Renováveis)/ICMBio (Instituto Chico Mendes de Conservação da Biodiversidade) license 19037−1 and by the Fiocruz’s Institutional Animal Care and Use Committee under licenses LW81−12, L50-16 and LM-6/18. Experimental infection in mice was performed following procedures approved in CEUA-Fiocruz license L006/2019. Anesthesia and euthanasia were performed according to the Animal Care and Use Committee licenses, and these procedures are cited in the first paragraph of the “Field procedures” section. All procedures were performed according to biosafety standards [39], and no environmental damage occurred.

## Results

A total of 270 bat individuals from 24 species and three families were collected in the four sampling areas, 75 individuals being in Peridomicile (A1), 94 in Initial Secondary Forest (A2), 39 in Late Secondary Forest (A3), and 62 in Mature Forest (A4) (Table 2). A total of 65 individuals from 15 species were positive for trypanosomatids in at least one of the examined tissues, resulting in an infection rate of 24.1%.

**Table 2:** Bats captured in four areas: Peridomicile—A1, Initial Secondary Forest —A2, Late Secondary Forest —A3, Mature Forest—A4 at Fiocruz Atlantic Forest Biological Station, Rio de Janeiro (RJ), Brazil, and examined for trypanosomatid infections.

Bat species	Infected/ Examined	A1	A2	A3	A4
*Anoura caudifer*	0/1 (0%)	–	–	–	0/1
*Artibeus cinereus*	1/3 (33.3%)	–	0/2	1/1	–
*Artibeus fimbriatus*	1/15 (6.7%)	1/6	0/2	0/2	0/5
*Artibeus lituratus*	16/81 (19.8%)	4/18	7/29	3/18	2/16
*Artibeus obscurus*	0/4 (0%)	0/1	0/2	–	0/1
*Carollia perspicillata*	15/61 (24.6%)	2/14	9/32	2/7	2/8
*Chiroderma villosum*	0/1 (0%)	0/1	–	–	–
*Desmodus rotundus*	2/23 (8.7%)	2/11	0/11	0/1	–
*Glossophaga soricina*	0/6 (0%)	0/6	–	–	–
*Glyphonycteris sylvestris*	0/1 (0%)	–	–	0/1	–
*Lasiurus blossevillii*	0/2 (0%)	–	–	0/2	–
*Micronycteris microtis*	0/2 (0%)	0/1	0/1	–	–
*Micronycteris minuta*	2/3 (66.7%)	–	2/2	0/1	–
*Mimon bennettii*	2/2 (100%)	–	1/1	1/1	–
*Molossus molossus*	0/7 (0%)	0/7	–	–	–
*Myotis izecksohni*	1/1 (100%)	–	–	–	1/1
*Myotis nigricans*	10/13 (76.9%)	3/3	–	–	7/10
*Myotis riparius*	6/11 (54.5%)	–	0/1	–	6/10
*Phyllostomus hastatus*	1/4 (25%)	–	1/4	–	–
*Plathirrynus recifinus*	1/1 (100%)	1/1	–	–	–
*Platyrrhinus lineatus*	0/2 (0%)	–	0/1	–	0/1
*Sturnira lilium*	2/12 (16.7%)	0/5	0/1	–	2/6
*Tonatia bidens*	3/9 (33.3%)	0/1	1/3	1/2	1/3
*Vampyressa pusilla*	2/5 (40%)	–	2/2	0/3	–
24 species	65/270 (24.1%)	13/75 (17.3%)	23/94 (24.5%)	8/39 (20.5%)	21/62 (33.9%)

Regarding the sampled areas and considering both parasitological and molecular diagnosis, 13 positive individuals were found in A1 (prevalence 17.3%; N = 75), 23 in A2 (24.5%; N = 94), 8 in A3 (20.5%; N = 39) and 21 in A4 (33.9%; N = 62). No significant differences (χ² = 4.01; p = 0.261) were detected among areas under the present sampling design.

### Parasitological diagnosis and culture characterization

Due to the low amount of blood that can be obtained from bats and the difficulties inherent to field logistics, fresh blood examination was performed for 185 individuals (68.5%; N = 270), and six of them (3.2%) were positive for flagellates’ presence. Of these, four were positive in other diagnostic tests and the other two were considered infected by trypanosomatids, without species characterization (S1 Table).

Considering the hemocultures, 23 individuals (8.5%; n = 270) were positive, and 22 isolates were obtained and cryopreserved. The molecular characterization revealed infection by: *T. dionisii* (n = 15; 68.2%), *C. mellificae* (n = 5; 22.7%), *T. cruzi* DTU TcI (n = 1; 4.5%) and *T. cruzi* DTU TcII (n = 1; 4.5%). Parasite populations from two culture sediments were characterized as *T. dionisii* and *T. cruzi* DTU TcI. In one of these cases, two characterizations were done for the same bat individual: the primary hemoculture sediment was characterized as *T. cruzi* DTU TcII, while the culture obtained from the re-isolation of the experimentally infected mouse was characterized as *T. cruzi* DTU TcI, pointing to a mixed *T. cruzi* infection (DTUs TcI and TcII) in this bat specimen (Table 3). No positive cultures were obtained from spleen, skin or liver tissues. The complete characterization of bat parasites, including collection site and taxonomy of the host, positive tissue, methodology of detection, GenBank access number, and COLTRYP catalogue number is presented in S1 Table.

**Table 3:** Bats infected by trypanosomatids in parasitological/molecular assays at EFMA, Rio de Janeiro (RJ), Brazil.

Collection Environment	Trypanosomatids Positive in Hemocultures	Trypanosomatids Positive in Molecular Diagnosis Directly on Tissues
A1 = 11 A2 = 23 A3 = 08 A4 = 21	*C. mellificae* (5 Isolates) *T. cruzi* DTU TcI (2 Isolates) *T. cruzi* DTU TcII (1 Sediment) *T. dionisii* (15 Isolates/1 Sediment)	*T. lainsoni* (3 Spleen) Trypanosomatidae (1 Liver/2 Spleen) *T. cruzi* DTU TcII (3 Liver/1 Spleen) *T. dionisii* (16 Liver/9 Skin/14 Spleen) *T.* sp. Neobat 1 (1 Liver/1 Skin/2 Spleen) *T.* sp. Neobat 4 (1 Liver/1 Spleen) *T. rangeli* A (1 Liver)
N = 63	N = 22 Isolates N = 2 Sediments	N = 23 Liver N = 10 Skin N = 23 Spleen

### Molecular diagnosis in bat tissues

From the 810 samples of tissue fragments (spleen, skin and liver: 270 each) molecularly evaluated, 56 samples (6.9%) were considered positive for trypanosomatid infection and characterization were performed in skin (n = 10; 3.7%), spleen (n = 23; 8.5%) and liver (n = 23; 8.5%) tissues (Table 3, S1 Table). Fifteen individuals had more than one tissue positive for trypanosomatid infection, and in only one of them, it was observed different trypanosomatid identifications: *T. lainsoni* in spleen and *T. dionisii* in liver (S1 Table).

The 72 sequences that were generated in this study are openly available on GenBank database (https://www.ncbi.nlm.nih.gov/genbank/) (accessed on 14 October 2025). Access numbers for 18S rDNA are provided in S1 Table. All raw diagnostic outcomes from parasitological culture and tissue-based PCR analyses are provided in S1 File.

**Fig 2:**
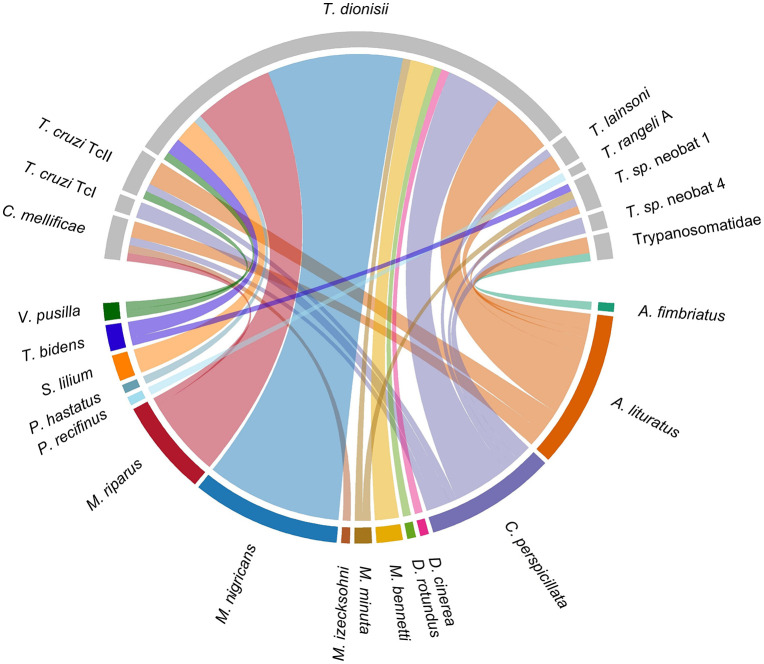
Chord diagram with bat species infected by different trypanosomatid species in EFMA. The image was generated in R (version 4.5.2) using the circlize package [40].

It was noted that the parasite *T. dionisii* was the most detected in this study compared to other trypanosomatids, mainly in bats of the species *M. nigricans*, *M. riparus*, *A. lituratus* and *C. perspicillata*. Probably as the result of being the most captured species, *A. lituratus* and *C. perspicillata* had the highest number of individuals considered positive for at least one trypanosomatid species, as shown in Fig 2.

**Fig 3:**
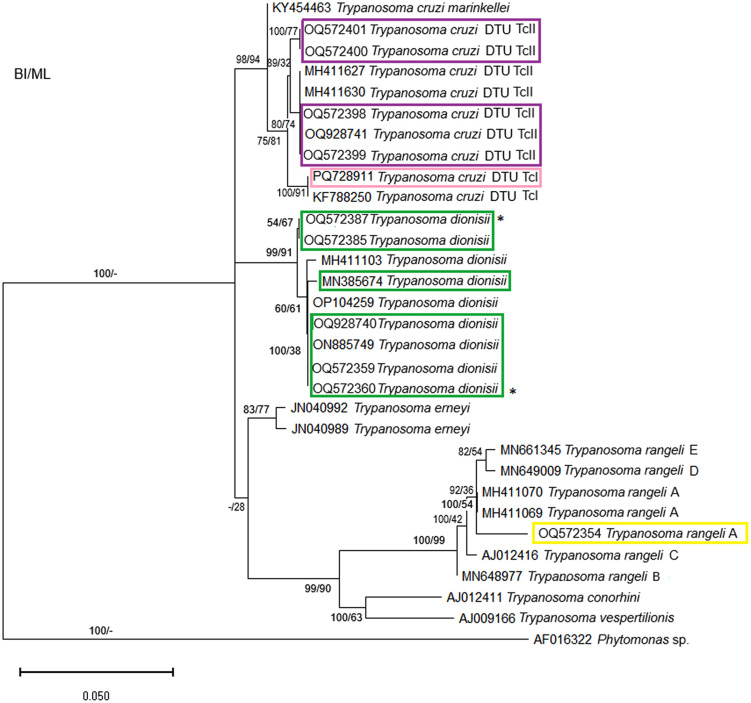
Phylogenetic analysis of 18S rDNA gene sequences indicates the phylogenetic position of trypanosomatids characterized as *T. cruzi* DTU TcII, *T. dionisii* and *T. rangeli* A, the samples OQ572387 and OQ572360 (*T. dionisii*) sinalized with * obtained 99% identity and coverage in GenBank. *Phytomonas* sp. was used as an outgroup.

Representative sequences of trypanosomatids species identified in the presented study that belong to *T. cruzi* clade are highlighted in Fig 3: *T. cruzi* DTU TcI (pink), *T. cruzi* DTU TcII (purple), *T. dionisii* (green) and *T. rangeli* A (yellow). Of the thirty-nine samples characterized as *T. dionisii*, seven representatives were used in construction of phylogenetic tree.

**Fig 4:**
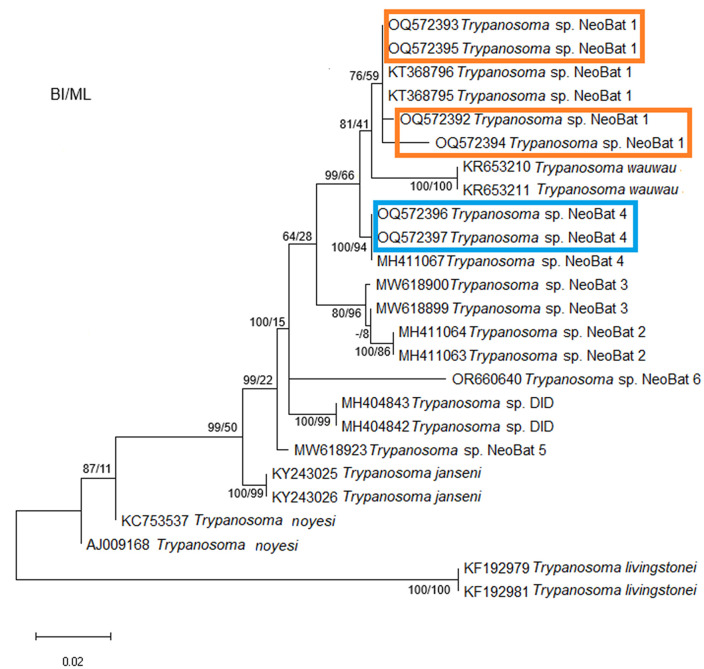
Phylogenetic analysis of 18S rDNA gene sequences indicates the phylogenetic position of trypanosomatids characterized as *T*. sp. Neobat 1 and 4. *Trypanosoma livingstonei* was used as an outgroup.

**Fig 5:**
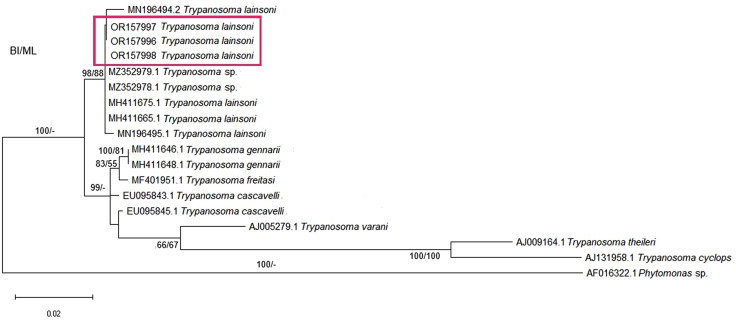
Phylogenetic analysis of 18S rDNA gene sequences indicates the phylogenetic position of trypanosomatid characterized in this study as *T. lainsoni.* *Phytomonas* sp. was used as an outgroup.

*Trypanosoma wauwau* subclade, although also within *T. cruzi* DTU clade is presented apart to demonstrate the phylogenetic position of the trypanosomatids characterized as *T.* sp. Neobat 1 (orange) and 4 (blue) (Fig 4). The phylogenetic position of the parasites characterized as *T. lainsoni* (pink) is demonstrated with the Trypanosomatids of reptile and mammal’s clade in Fig 5.

## Discussion

The results indicated that, despite the conspicuous differences in habitat structure, there were no statistically significant differences in the infection rates (prevalence) of trypanosomatids in bats from the four sampled areas. The slightly higher diversity of trypanosomatid species detected in A2 can be explained by the largest number of bat individuals collected. Thus, although there are conspicuous differences in habitat structure, these attributes are not affecting the rates of bat infection by trypanosomatids. This result may be related to three factors: (i) bats are recognized habitat-resilient mammals; (ii) proximity between the sampled areas (< 2 km), and (iii) higher positive rates for common bat species (*i.e.*, *Carollia perspicillata*, *Artibeus lituratus*, and *Myotis nigricans*). These species are among the most abundant in the Brazilian Atlantic Forest, including EFMA, occupying a wide variety of habitats, from pristine forests to densely urbanized areas, being well adapted to the anthropic habitat disturbances [25,41]. In addition, some of these species have large home ranges and realize long-distance movements [42,43]. Thus, the distance between sampled areas and the difference in habitat structure does not represent barriers to these bats, which can promote the dispersion of tripanosomatids throughout the EFMA’s landscape, considering that the biological vectors of those infections are also dispersed in the four areas. These hypotheses are corroborated by an earlier study conducted in Atlantic Forest locations with different levels of anthropization that also found no differences in bat infection by trypanosomatids between urban and continuous forest sites [5].

It was possible to isolate parasites in the hemocultures from more than one third of the infected bats (35.4%; n = 23/65), a success higher than those observed for marsupials and rodents (27.7%; N = 25/90) [19] and even bats (10,8%; N = 7/65) [5] examined in previous studies conducted in the EFMA using similar methodology. The positive hemocultures indicate high parasitemia and, consequently, that these animals may act as sources of infection for vectors at the time of capture. This was observed for *T. dionisii,* a parasite related to bats that was previously demonstrated to circulate in this area, *T. cruzi* DTU TcI, also reported in bats and small mammals in EFMA [5,19], but also *Crithidia mellificae¸* classically considered as monoxenous parasite associated to bees that was first described in a bat *Anoura caudifer* from Guapiaçu Ecological Reserve, another Atlantic Forest protected area from Rio de Janeiro state) [5] and also recently described in EFMA [2].

The bat species *A. lituratus* and *C. perspicillata* had the largest number of individuals collected and had the highest positivity rates for trypanosomatid infections. Among these infections are *T. dionisii*, *T. lainsoni*, *Trypanosoma* sp. Neobat 1 and *C. mellificae* that were detected in both bat species. Differently, *T. cruzi* DTU TcII was detected in *A. lituratus,* while *T. cruzi* DTU TcI and *T.* sp. Neobat 4 were detected in *C. perspicillata*. The generalist habits of both species probably expose them to a high diversity of trypanosomatid species in distinct transmission cycles that occur in the most diverse types of habitats.

The importance of including the molecular diagnosis directly in tissues is demonstrated by the detection of parasites reported as unable to grow in the usually employed culture media (NNN with LIT or Schnneider), as is the case of *Trypanosoma* sp. Neobats [10–13]. An important aspect to be highlighted is that none of the tissues positive in PCR were positive in culture, even when it was detected parasite species that are usually isolated in blood, such as *T. rangeli*, *T. cruzi* and *T. dionisii.* This can be explained by the shorter follow-up period of the cultures (one month), compared to cultures from blood samples that are followed up for up to three to five months. This shorter follow-up is related to the fact that tissues cultures were performed mainly to isolate *Leishmania* parasites, whose generation time is shorter than *Trypanosoma*, and because of the impossibility of long-term monitoring due to bacterial contamination of the tissue cultures, usually more frequent and more intense than that observed in hemoculture. Another possible reason is that these bats whose tissues were positive only by PCR were not in the early stages of infection, when parasitemia were already controlled and the parasite load was low. Notably, the use of viscera DNA in molecular diagnosis for trypanosomatids was confirmed to be useful in parasitological studies, as previously seen in other studies [19,44]. This is especially important for spleen and liver tissues, which are organs that filter the blood of host organism, where the DNA of hemoparasites, like trypanosomatids, can be present in large quantities and be detected molecular assays, resulting in a higher diversity of parasites that could be underestimated in parasitological assays or other tissues.

Finally, we cannot rule out that the absence of parasite isolation is related to a low parasite load in tissues, that enables molecular detection, but not parasite isolation. This is the probable explanation for the high number of *T. dionisii* positive samples, including hosts that were positive for this same parasite in more than one tissue. In fact, *T. dionisii* is a parasite species frequently reported in bats with different feeding habits, including insectivorous, frugivorous and hematophagous species, which was already be related to genetic heterogeneity [14]. Not surprisingly, this was the unique parasite species detected in the four evaluated areas, confirming its wide distribution throughout the EFMA, despite the higher detection in A4, the most preserved area. Although *T. dionisii* was considered as restricted to bats for several decades, recent studies refuted this assumption by demonstrating the infection in humans, marsupials, rodents, and carnivores [13,15,16,19,45].

It is worth mentioning that only one bat individual (LBT 10363) was detected with mixed infection: *T. dionisii* in the liver and *T. lainsoni* in the spleen. Mixed infections are commonly found in studies with wild hosts, such as bats that are known hosts of several species of trypanosomatids [46]. But herein, it was not observed in all other fourteen bat individuals that were positive in more than one tissue, and whose identifications were always the same parasite species, in these cases, *T. dionisii*. This data reflects the wide parasite distribution in different tissues of an infected bat, highlighting once again the association of *T. dionisii* with the order Chiroptera, which has been extensively described in the literature for years [5,13–15].

This study detected for the first time in EFMA the infection by *T. lainsoni* in spleen samples from bats (two *A. lituratus* and one *C. perspicillata*). This parasite is commonly reported in rodents but was also previously detected in other bat species (*Artibeus planirostris* and *Platyrrhinus lineatus*), besides in individuals from Didelphimorphia and Carnivora orders [13,47,48]. From the three *T. lainsoni* infected bats, one of them demonstrated to be co-infected with *T. dionisii* (LBT 10363), demonstrating the participation of bats from EFMA in the overlapped life cycles of both *Trypanosoma* species. Mixed infections are commonly found in bat species, and this may be related to the ability of their immune system to tolerate several types of infections from different parasite species simultaneously, as previously described [13,15].

*Trypanosoma cruzi* infection was found in six bat individuals, which one of them presented mixed infection by two *T. cruzi* DTUs, TcI and TcII. The mixed initial inoculum contained both DTU TcI and DTU TcII subpopulation, but only the first was selected after mouse infection, as a result of the recognized possibility that any host can act as biological filter for distinct parasite subpopulations [49]. *Trypanosoma cruzi* DTU TcI was isolated from blood of another bat individual, while *T. cruzi* DTU TcII was detected directly in tissues from four individuals (three in liver and one in spleen). This is the first report of DTU TcII in the EFMA, because previous studies have reported so far only the DTUs TcI, TcIII and TcIV [5,19]. Considering the ubiquitous nature of this DTU, the lack of previous records of this DTU is probably due to the lack of representative studies of the local fauna.

*Trypanosoma rangeli* A was found in a liver sample from a *Platyrrhinus recifinus* captured in A1, and this is the second report of this genotype in Atlantic Forest, more precisely in EFMA. Previously, *T. rangeli* A was isolated from a *Didelphis aurita* in the same sampled area, A1 [19]. As stated by Dario *et al*. [50], the distribution of this subpopulation is wider than observed for other *T. rangeli* subpopulations.

*Trypanosoma* sp. Neobat 1 and Neobat 4 were detected in spleen, liver and skin tissues from six individuals. These parasites are part of *Trypanosoma* clade spp. Neobats, and until now, six distinct genotypes, named from 1 to 6, are recognized [10–12]. Apparently, these parasites do not grow in the most employed axenic culture medium (NNN with LIT or Schnneider) and, consequently, isolates from *Trypanosoma* sp. Neobat are not available, and nothing is known about their morphology or life cycle [10]. In this study, *T.* sp. Neobat 4 was found in two individuals of *C. perspecillata*. This infection is commonly found in *C. perspecillata* and was previously proposed as specific for this bat species [11]. Recently, however, this hypothesis was refuted because this genotype parasite was detected in *Carollia sowelli* and *Choeroniscus godmani* from Mexico, pointing to the versatility and capacity of adapting to other bat host species [10].

The taxonomic identification of the trypanosomatids were also confirmed by their phylogenetic position in the respective trees containing reference sequences available in GenhBank and the sequences herein obtained. In this sense, the phylogenetic trees of the *T. cruzi* clade confirmed the different *T. cruzi* DTUs detected besides the characterization of *T. rangeli* as subpopulation A and the identification of *T. dionisii* infections. The second phylogenetic tree, referring to *T. wauwau* subclade that is part *T. cruzi* clade, indicated and confirmed the phylogenetic position of the NeoBats detected in this study, which were defined as belonging to genotypes 1 and 4. The third phylogenetic tree, referring to a clade named Trypanosomatids from reptiles and mammals, confirmed the phylogenetic position and characterization of *T. lainsoni,* a parasite up to now detected only in mammals.

*Crithidia mellificae* is considered a monoxenic trypanosomatid that infects insects. However, several reports of this infection in different groups of mammals, such as species of the Chiroptera, have already been described [2,5]. In this study, infection by this parasite was detected in five individuals, all through isolation in blood, as previously reported by Dario *et al*. [2].

Besides the two individuals that were positive in the fresh blood examination, but negative in the molecular assays; three individuals were considered positive for trypanosomatid infection, in spleen or liver tissues. Taxonomic characterization at the species level was not achieved, even after attempts at reamplification and subsequent sequencing, where the amplified DNA always presented weak and/or nonspecific bands in the 2% agarose gel, and the sequencing of these samples also presented electropherograms with very high and/or very low peaks; and thus, for this reason, they were named as Trypanosomatidae. Even if characterization at the species level was not achieved in these three cases, it is important to highlight the success in molecular identification of the infection directly in viscera, demonstrating the suitability of this method in detecting trypanosomatid infection in the lack of parasite isolation.

The study has limitations that prevent us in making ecological inferences about patterns involving the parasite species found with a particular bat species or capture area. All interpretations made in the manuscript are clearly framed as descriptive rather than inferential due to the study design limitation. Ecological implications are also not measurable given the high longevity of these animals, their mobility, and the lack of studies describing the consequences of *Trypanosoma* sp. infections on bats’ health, especially because for many of the species and genotypes found in this study, we lack information on their biological aspects and even vectors involved. However, the epidemiological implications and the possibility of parasite exchange were discussed. Although we carried out a significant capture effort of 36 nights homogeneously distributed across four areas, it was not homogeneously in terms of temporal distribution. Furthermore, the use of only one capture method (mist-nets no higher than three meters for four hours) does not allow the capture of bats that fly at higher altitudes or at times closer to dawn. For these reasons, our analyses focused on identifying parasites infecting bats, rather than on ecological analyses of hosts or parasites.

## Conclusion

This study demonstrated that the molecular diagnosis performed directly in viscera was efficient to detect a remarkable richness of trypanosomatid species and/or genotypes, including *Trypanosoma* sp. Neobat 1 and 4, pointed as non-cultivable in the usually employed axenic media. Besides *Trypanosoma rangeli* and *T. dionisii*, this study demonstrated for the first time the circulation of *T. cruzi* DTU TcII and *T. lainsoni* in EFMA. It was possible to isolate *T. dionisii, T. cruzi* DTU TcI and *C. mellificae* from bats’ blood, pointing these flying mammals as potential sources of infection for vectors in this area. Furthermore, *T. dionisii* was also detected in different bat species, indicating that this parasite is well dispersed in EFMA areas. The bat species *A. lituratus* and *C. perspicillata* had the highest number of individuals infected by trypanosomatids, probably as the result of their generalist habits. The high diversity of trypanosomatid species and/or genotypes infecting bats in the small and human-impacted forest remnant from EFMA reinforces the complexity of those parasites sylvatic transmission that may not be disrupted even under strong anthropogenic pressures.

## Supporting information

S1 Table Bats infected by trypanosomatids in parasitological/molecular assays at EFMA, Rio de Janeiro (RJ), Brazil, between 2013, 2014, 2016–2019. The bats are identified by taxonomic species, sample ID and collection environment. The infections are presented separately (if hemoculture or molecular diagnosis directly on tissues) with their respective GenBank access number and COLTRYP catalogue number.
(DOCX)

S1 File Raw data of registration number, collection date, bat species, Collection area and results of fresh blood examination, hemoculture and the respective Coltryp number (when positive), tissue cultures and the results of the molecular characterization obtained for each positive tissue. (XLSX)
