# Supplementary material for: Trypanosomatid species infecting bats in the Fiocruz Atlantic Forest Biological Station, an urban forested fragment in Rio de Janeiro, Brazil
Source: PLoS One. 2026 May 18;21(5):e0349312. doi: 10.1371/journal.pone.0349312 (PMC13183226; doi:10.1371/journal.pone.0349312)
Supplement: S1 Table — The bats are identified by taxonomic species, sample ID and collection environment. The infections are presented separately (if hemoculture or molecular diagnosis directly on tissues) with their respective GenBank access number and COLTRYP catalogue number. (DOCX) [file pone.0349312.s001.docx]

**S1 Table. Bats infected by trypanosomatids in parasitological/molecular assays at EFMA, Rio de Janeiro (RJ), Brazil, between 2013, 2014, 2016 - 2019.** The bats are identified by taxonomic species, sample ID and collection environment. The infections are presented separately (if hemoculture or molecular diagnosis directly on tissues) with their respective GenBank access number and COLTRYP catalogue number.

| **Collection Environment** | **Sample ID** | **Species** | **Hemocultures** | **Tissues** | **GenBank Access Number** | **COLTRYP Number** |
| --- | --- | --- | --- | --- | --- | --- |
| A1 | RM 452 | *Myotis nigricans* | Neg | *T. dionisii* (L) | OQ572355 | - |
|  | RM 462 | *Myotis nigricans* | Neg | *T. dionisii* (Sp, L) | OQ572356 (Sp)/ OQ572357(L) | - |
|  | RM 463 | *Desmodus rotundus* | Neg | *T. dionisii* (Sp) | OQ572358 | - |
|  | RM 465 | *Carollia perspicillata* | *T. dionisii* | Negative | OQ418069 | C00635 |
|  | RM 481 | *Artibeus lituratus* | Neg | Trypanosomatidae (Sp) | - | - |
|  | RM 565 | *Plathirrynus recifinus* | Neg | *T. rangeli* A (L) | OQ572354 | - |
|  | LBT 10381 | *Artibeus lituratus* | Neg | *T. cruzi* DTU TcII (Sp) | OQ572400 | - |
|  | LBT 10382 | *Artibeus lituratus* | Neg | *T. dionisii* (S) | OQ572387 | - |
|  | LBT 10383 | *Artibeus lituratus* | Neg | *T. dionisii* (S) | OQ572384 | - |
|  | LBT 10850 | *Artibeus fimbiatrus* | Neg | Trypanosomatidae (L) | - | - |
|  | LBT 10860 | *Myotis nigricans* | Neg | *T. dionisii* (Sp, L) | OQ572369 (Sp)/ OQ572370 (L) | - |
| A2 | RM 438 | *Carollia perspicillata* | Neg | *T.* sp*.* Neobat 4 (L) | OQ572396 | - |
|  | RM 468 | *Carollia perspicillata* | Neg | *T.* sp*.* Neobat 4 (Sp) | OQ572397 | - |
|  | RM 469 | *Carollia perspicillata* | *T. dionisii* | Negative | KY689927 | C00543 |
|  | RM 491 | *Mimon bennettii* | Neg | *T.* sp. Neobat 1 (Sp) | OQ572392 | - |
|  | RM 493 | *Artibeus lituratus* | Neg | *T.* sp. Neobat 1 (Sp) | OQ572393 | - |
|  | RM 541 | *Micronycteris minuta* | *T. dionisii* | *T. dionisii* (L) | KY689931/ OQ572361(L) | C00570 |
|  | RM 578 | *Artibeus lituratus* | Neg | Trypanosomatidae (Sp) | - | - |
|  | LBT 9734 | *Carollia perspicillata* | *T. dionisii* | Negative | ON980675 | C00767 |
|  | LBT 9735 | *Carollia perspicillata* | *T. dionisii* | Negative | ON980676 | C00768 |
|  | LBT 10362 | *Phyllostomus hastatus* | Neg | *T. dionisii* (S) | OQ572385 | - |
|  | LBT 10363 | *Artibeus lituratus* | Neg | *T.* *lainsoni* (Sp)/ *T. dionisii* (L) | OR157998 (Sp) /OQ572382 (L) | - |
|  | LBT 10364 | *Carollia perspicillata* | Neg | *T. lainsoni* (Sp) | OR157997 | - |
|  | LBT 10368 | *Artibeus lituratus* | Neg | *T. dionisii* (S, L) | OQ572382 (S)/ OQ572389 (L) | - |
|  | LBT 10369 | *Carollia perspicillata* | Neg | *T. dionisii* (S) | OQ572386 | - |
|  | LBT 10370 | *Artibeus lituratus* | Neg | *T. dionisii* (S) | OQ572388 | - |
|  | LBT 10371 | *Carollia perspicillata* | *T. cruzi* DTU TcI² | Negative | - | C00811 |
|  | LBT 10372 | *Artibeus lituratus* | Neg | *T. dionisii* (S) | OQ572391 | - |
|  | LBT 10373 | *Vampyressa pusilla* | Neg | *T. cruzi* DTU TcII (L) | OQ572401 | - |
|  | LBT 10374 | *Vampyressa pusilla* | Neg | *T. dionisii* (Sp) | OQ572390 | - |
|  | LBT 10375 | *Micronycteris minuta* | *T. dionisii*¹ | Negative | OQ928740¹ | - |
|  | LBT 10840 | *Tonatia bidens* | *T. dionisii* | Negative | ON885749 | C00831 |
|  | LBT 11317 | *Artibeus lituratus* | Neg | *T. dionisii* (Sp) | OQ572380 | - |
|  | LBT 11323 | *Carollia perspicillata* | *T. dionisii* | Negative | OP265720 | C00861 |
| A3 | RM 436 | *Carollia perspicillata* | Neg | *T.* sp. Neobat 1 (L) | OQ572394 | - |
|  | RM 500 | *Dermanura cinerea* | Neg | *T. dionisii* (Sp) | OQ572359 | - |
|  | RM 501 | *Mimon bennettii* | Neg | *T. dionisii* (L) | OQ572360 | - |
|  | LBT 10096 | *Tonatia bidens* | Neg | *T.* sp. Neobat 1 (S) | OQ572395 | - |
|  | LBT 10393 | *Artibeus lituratus* | Neg | *T.* *lainsoni* (Sp) | OR157996 | - |
|  | LBT 10869 | *Artibeus lituratus* | *C. mellificae*³ | Negative | MN879778 | C00821 |
|  | LBT 10872 | *Artibeus lituratus* | *C. mellificae*³ | Negative | MN879777 | C00820 |
|  | LBT 10873 | *Carollia perspicillata* | *C. mellificae*³ | Negative | MN879776 | C00818 |
| A4 | LBT 8706 | *Myotis riparus* | Neg | *T. dionisii* (Sp, L) | OQ572375 (Sp) /OQ572371 (L) | - |
|  | LBT 8707 | *Myotis nigricans* | Neg | *T. dionisii* (Sp, L) | OQ572363 (Sp) /OQ572372 (L) | - |
|  | LBT 8708 | *Myotis riparus* | Neg | *T. dionisii* (Sp, L) | OQ572364 (Sp)/ OQ572373 (L) | - |
|  | LBT 8709 | *Myotis riparus* | Neg | *T. dionisii* (Sp, L) | OQ572365 (Sp)/  OQ572374 (L) | - |
|  | LBT 8997 | *Myotis nigricans* | Neg | *T. dionisii* (Sp, L) | OQ572377 (Sp)/ OQ572376 (L) | - |
|  | LBT 8999 | *Carollia perspicillata* | *T. dionisii* | Negative | MN385674 | C00737 |
|  | LBT 9000 | *Myotis nigricans* | Neg | *T. dionisii* (Sp, L) | OQ572378 (Sp)/ OQ572379 (L) | - |
|  | LBT 9045 | *Myotis nigricans* | *T. dionisii* | *T. dionisii* (Sp)² | MN385679 | C00742 |
|  | LBT 9046 | *Tonatia bidens* | *T. dionisii* | Negative | MN385682 | C00745 |
|  | LBT 9050 | *Myotis nigricans* | *T. dionisii* | Negative | MN385680 | C00743 |
|  | LBT 9051 | *Myotis nigricans* | *T. dionisii* | Negative | MN385681 | C00744 |
|  | LBT 10082 | *Artibeus lituratus* | Neg | *T. cruzi* DTU TcII (L) | OQ572398 | - |
|  | LBT 10084 | *Myotis nigricans* | *T. dionisii* | *T. dionisii* (L) | ON980681/ OQ572362(L) | C00788 |
|  | LBT 10086 | *Artibeus lituratus* | Neg | *T. cruzi* DTU TcII (L) | OQ572399 | - |
|  | LBT 10395 | *Sturnira lilium* | *T. dionisii* | *T. dionisii* (Sp) | OP265718/ OQ572381 (Sp) | C00814 |
|  | LBT 10399 | *Carollia perspicillata* | *T. cruzi* DTU TcI/ *T. cruzi* DTU TcII¹ | Negative | PQ728911/  OQ928741¹ | C00817 |
|  | LBT 10868 | *Myotis riparus* | *C. mellificae*³ | Negative | MN879775 | C00819 |
|  | LBT 10874 | *Myotis riparus* | Neg | *T. dionisii* (S, Sp, L) | OQ572368 (S) /OQ572367(Sp)/PQ474670 (L) | - |
|  | LBT 10875 | *Sturnira lilium* | *T. dionisii* | Negative | ON885747 | C00823 |
|  | LBT 10878 | *Myotis riparius* | Neg | *T. dionisii* (S) | OQ572366 | - |
|  | LBT 10879 | *Myotis izecksohni* | *C. mellificae³* | Negative | MN879779 | C00822 |
| A1= 11  A2= 23  A3= 8  A4= 21 | N = 63 | 16 species | *C. mellificae* (5)  *T. cruzi* DTU TcI (2)  *T. cruzi* DTU TcII (1)  *T. dionisii* (16) | *T. lainsoni* (3)  Trypanosomatidae (3)  *T. cruzi* DTU TcII (4)  *T. dionisii* (39)  *T.* sp. neobat 1 (4)  *T.* sp. neobat 4 (2)  *T. rangeli* A (1) | Sequences (72) | Isolates  (22) |

A1, Peridomicile; A2, Initial Secundary Forest; A3, Late Secundary Forest; A4, Mature Forest; (S) skin; (Sp) Spleen; (L) liver. Neg (Negative). ¹Sediment (characterized in the culture re-isolated from the experimentally infected mouse). ²The sequence of this sample (LBT 10371) was not deposited in GenBank database, because this sequence was considered too short, with less than 300 base pairs to be deposited, and (LBT 9045 Sp) only the reverse strand could be sequenced even after repeating the sequencing process. ³Samples that have previously been published by Dario *et al*., 2021.
